# Dynamic Shift from CD85j/ILT-2 to NKG2D NK Receptor Expression Pattern on Human Decidual NK during the First Trimester of Pregnancy

**DOI:** 10.1371/journal.pone.0030017

**Published:** 2012-01-05

**Authors:** Romain Marlin, Marion Duriez, Nadia Berkane, Claire de Truchis, Yoann Madec, Marie-Anne Rey-Cuille, Jean-Saville Cummings, Claude Cannou, Heloise Quillay, Françoise Barré-Sinoussi, Marie-Thérèse Nugeyre, Elisabeth Menu

**Affiliations:** 1 Regulation of Retroviral Infection Unit, Department of Virology, Institut Pasteur, Paris, France; 2 Department of Gynecology Obstetrics and Reproductive Medicine, Tenon Hospital, AP-HP, UMPC, Paris, France; 3 Gynecology-Obstetrics Service, A. Béclère Hospital, AP-HP, Clamart, France; 4 Emerging Disease Epidemiology Unit, Department of Infection and Epidemiology, Institut Pasteur, Paris, France; University of Montreal, Canada

## Abstract

During the first trimester of human pregnancy, Natural Killer (NK) cells of the maternal uterine mucosa (e.g. decidua) have a unique phenotype and are involved in crucial physiological processes during pregnancy. We investigated whether modifications of the NK receptor repertoire occur during the first trimester of pregnancy. We found significantly decreased expression of KIR2DL1/S1 and KIR2DL2/L3/S2 receptors, NKp30 and NKp44 activatory receptors, and the CD85j (ILT-2) inhibitory receptor. We also observed significantly increased expression of the NKG2D activatory receptor at the decidual NK cell surface. By flow cytometry, we further highlighted an evolution of NK subsets between 8 and 12 weeks of gestation, with a shift from the KIR2DL1/S1^+^/KIR2DL2/L3/S2^+^ subset towards the double negative subset, coupled with a decrease of the CD85j^+^/NKG2D^−^ subset in favour of the CD85j^−^/NKG2D^+^ subset. Furthermore, cell surface expression of NK receptor ligands, including CD85j and NKG2D ligands, has been characterized by flow cytometry on decidual immune CD14^+^ and CD3^+^ cells. HLA-G, the high affinity ligand of CD85j, was detected on both cell types. In contrast, NKG2D ligands ULBP-2 ULBP-3 and MICA/B were not expressed on CD14^+^ and CD3^+^ cells, however a variable expression of ULBP-1 was observed. The ligand expression of KIR2DL1/S1 and KIR2DL2/L3/S2 was also analyzed: the HLA-C molecule was expressed at a low level on some CD14^+^ cells whereas it was not detected on CD3^+^ cell surface. NK receptor ligands are known to be also expressed on the invading placental trophoblast cells. Thus, the phenotypic evolutions of decidual NK cells described in this present study may preserve their activation/inhibition balance during the first trimester of pregnancy.

## Introduction

Natural killer (NK) cells are a component of the innate immune system, and play an important role in the initiation of an efficient immune response. They can eliminate pathogen-infected cells as well as transformed tumor cells, by using a broad panel of inhibitory and activatory receptors [1], [2]. The main NK receptors belong to the Ig-like superfamily (KIR, LILR, NCR) or the C-type lectin family (NKG2 receptors) [3]. These receptors recognize MHC class I molecules including HLA-A, -B, -C, -E and -G; but also stress-inducible proteins or viral proteins [4]. NK cells are widely present throughout the human body and are found in peripheral blood [5], the liver [6], the gut [7] and the female reproductive tract [8]. Moreover, these innate immune cells are involved in the pathogenesis of several infections. For example, NKG2D, CD85j (also known as ILT-2, LILRB-1 and LIR-1) and NKp44, among other NK receptors, appear to be crucial for mechanisms of control of Cytomegalovirus or Human Immunodeficiency Virus type 1 infections [9], [10], [11]. Studies reported during the last decade have shown that the roles of NK cells are not restricted to a killing function. Indeed evidence exists for regulatory and building activities, especially concerning uterine NK cells within the female reproductive tract [12].

Between 6 and 7 days after ovulation, the blastocyst-stage egg attaches to the uterine mucosa and invades the monostratified epithelium. This process induces the placental development. After the initial step of nidation, the differentiation of placental trophoblast cells follows two distinct pathways: villous and extravillous. During the first trimester of pregnancy, extravillous trophoblast cells (EVT) derived from the placenta progressively invade the maternal uterine mucosa (i.e. the decidua) and then the inner third of the myometrium.

During the first trimester of pregnancy, NK cells represent the most abundant immune cells in the decidua [13]. These decidual NK (dNK) cells are CD56^bright^/CD16^negative^ and are distinct from peripheral blood NK subsets [14]. At this time, the dNK cells are crucial for the maintenance of pregnancy and their functions mainly involve cell-to-cell contact and the secretion of a large panel of cytokines, chemokines and growth factors [15], [16]. dNK cells produce pro-angiogenic factors (IFN-γ, VEGF, Ang2 etc.), which stimulate the remodelling of the uterine spiral arteries [17]. These cells also secrete IL-8 and CXCL-10 that stimulate EVT cell invasion [18]. During this process, dNK cells are in close contact with EVT cells; however they do not kill these semi-allogenic cells [19]. Despite the fact that dNK cells contain large intracellular amounts of perforin and granzyme A, they do not release their cytolytic granules during a healthy pregnancy [20]. Moreover, dNK cell functions also involve interactions with other immune decidual cells, such as CD14^+^ antigen presenting cells and CD3^+^ T lymphocytes [21], [22]. While expression of dNK ligands on human trophoblast cells is well described [18], [19], [21], [23], less is known concerning their surface expression on immune decidual cells.

During pregnancy, the structure of the decidual mucosa is modified. The EVT cells invade the mucosa in depth [24]. Moreover, the immune composition of the decidual tissue evolves with time. After a massive recruitment or proliferation of NK cell subsets, the number of dNK cells decreases from the end of the first trimester until the end of pregnancy [25]. In addition to the change in the dNK cell proportion, the role of dNK cells is also modified during pregnancy. Studies have shown several functional differences between dNK cells at the end of the first trimester of pregnancy (8 to 10 weeks of gestation) and those of the second trimester (12 to 14 weeks of gestation) [26]. dNK cells secrete more angiogenic factors during the first trimester [17] and larger amounts of IL-6, IL-8 and IFN-γ in the second trimester [26]. Recently, it was shown that the first trimester dNK cells are much more capable of stimulating trophoblast invasion than the second trimester dNK cells [27].

In the present study, we investigated whether modifications of the NK repertoire at the surface of dNK cells occur during the first trimester of pregnancy. We showed that the expression of CD85j, NKG2D, NKp30, NKp44, KIR2DL1/S1 and KIR2DL2/L3/S2 receptors at the cell surface evolves between 8 and 12 weeks of gestation. We were able to characterize a strong association between the expression levels of several of these receptors and to demonstrate for the first time the dynamics of the CD85j/NKG2D dNK subsets. We also detected NK receptor ligands at the cell surface of decidual immune CD14^+^ and CD3^+^ cells. CD85j ligand (HLA-G) and ULBP-1, one of the ligand of NKG2D, were observed on both cell types. Others NKG2D ligands were not detected. Common ligand of KIR2DL1/S1 and KIR2DL2/L3/S2 (HLA-C) was only observed at a low level on few CD14^+^ cells. Previous data have shown that the invading placental trophoblast cells also express NK receptor ligands [19], [23], [28], [29]. Thus our results suggest that the dynamic expression of NK receptors may ensure the activation/inhibition balance of dNK cells during the trophoblast invasion process.

## Results

### Decidual NK cells express inhibitory and activatory receptors with varying donor-specific percentage of expression

**Figure 1:**
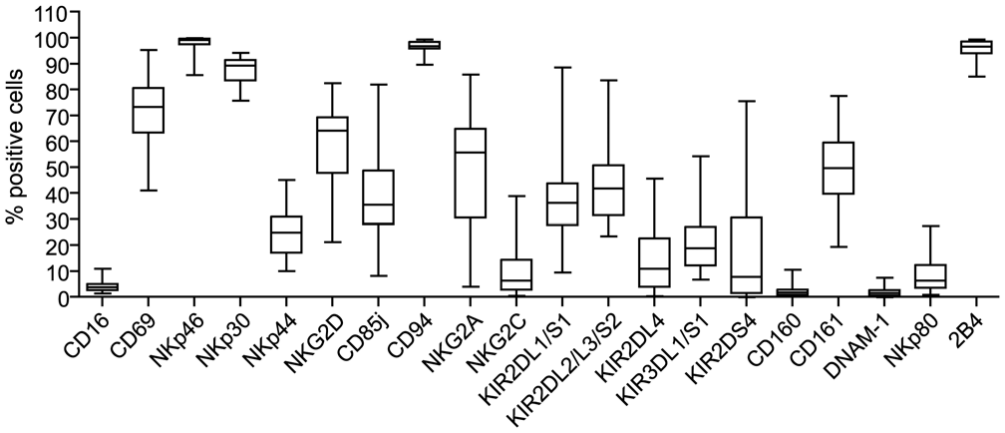
Expression of the NK receptor repertoire at the surface of decidual NK cells during the first trimester of pregnancy. Decidual mononuclear cells were analyzed by flow cytometry immediately after isolation. The analyses were gated on the CD56^+^/CD3^−^ population. Box-and-whisker plots represent percentages of positive cells for each receptor. Analyses were performed on decidual cells from 28 different donors between 8 and 12 weeks of gestation.

To characterize the dNK cell repertoire, we performed flow cytometry analysis of 20 NK receptors on freshly isolated decidual mononuclear cells (n = 28) (Fig. 1). We confirmed that dNK cells express the KIR which bind to HLA-C (KIR2DL1/S1, KIR2DL2/L3/S2 and KIR2DS4) but also express KIR3DL1/S1 and the KIR which binds to HLA-G, KIR2DL4. The NCR activatory receptors (NKp46, NKp30 and NKp44) and NKG2D were also detected at the dNK cell surface, however a few percentage of dNK cell express the NKG2C activatory receptor. dNK cells also expressed inhibitory receptors such as CD94/NKG2A and CD85j. The majority of dNK cells were positive for the 2B4 co-receptor and the CD69 activation marker. Moreover, we confirmed that dNK cells did not express the CD16 and CD160 receptors at their surface. We showed for the first time that the CD161 and NKp80 receptors were expressed at the dNK surface at a high and low percentage, respectively. Furthermore we also established that the DNAM-1 receptor is not expressed.

Importantly, we observed variations in NK receptor cell surface expression between donor samples for the majority of NK receptors; with the exception of CD16, NKp46, CD94, CD160, DNAM-1, and 2B4 (Fig. 1). Since the samples were obtained between 8 and 12 weeks of gestation, these variations might be related to time of gestation.

### Expression of NK receptors on the dNK cells evolves during the first trimester of pregnancy

**Figure 2:**
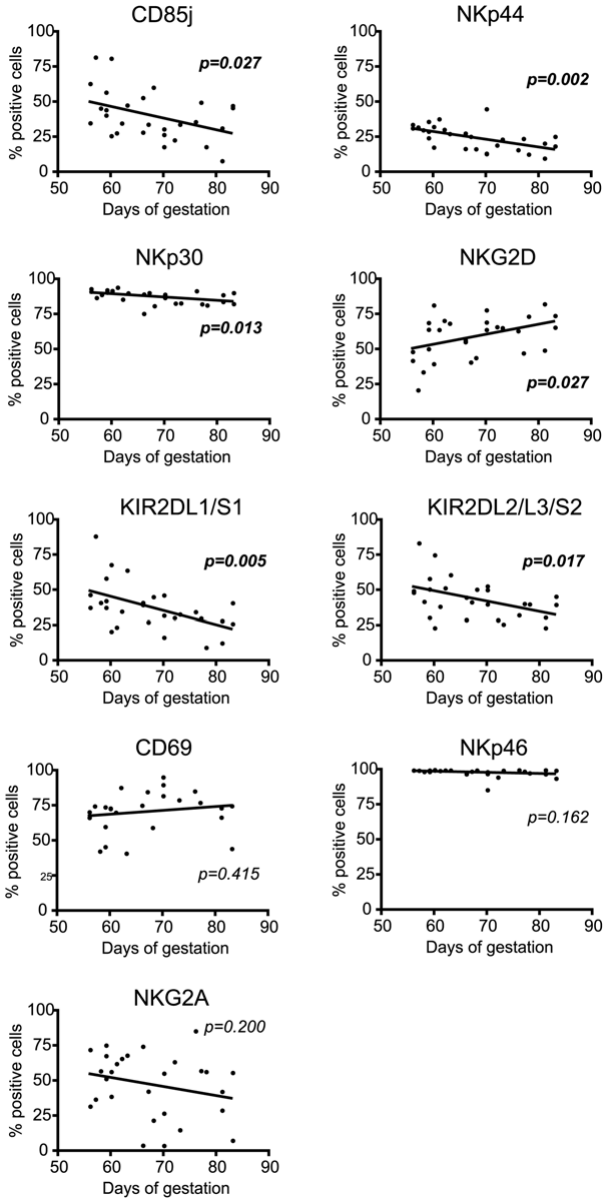
NK receptor repertoire evolution of dNK cells during the first trimester of pregnancy. Expression of NK receptors on decidual mononuclear cells was analyzed between 8 and 12 weeks of gestation. Graphs represent the percentage of marker expression (Y) and the day of gestation (X). The line represents the linear regression. A p-value<0.05 was significant. The analyses were performed on decidual cells from 28 different donors, individually represented by a dot.

To assess whether the expression of NK receptors was modified during the first trimester of pregnancy, we analyzed the surface expression of each of the 20 studied NK receptors between 8 and 12 weeks of gestation on freshly isolated decidual mononuclear cells (n = 28) (Fig. 2). Using a linear regression test, we observed a significant decrease in the surface expression of the CD85j inhibitory receptor over this period (p = 0.027). Moreover the extent of surface expression of the NKp30 and NKp44 activatory receptors also decreased significantly (p = 0.013 and p = 0.002 respectively), whereas expression of the NKG2D activatory receptor at the dNK cell surface was significantly increased (p = 0.027) over the same gestational period. As previously reported [30], the KIR2DL1/S1 and KIR2DL2/L3/S2 KIR receptors were significantly decreased between 8 and 12 weeks of gestation (p = 0.005 and p = 0.017 respectively). In contrast, the expression of CD69, NKp46, NKG2A (Fig. 2), NKG2C, CD94, 2B4, KIR2DL4, KIR3DL1/S1, KIR2DS4, NKp80 and CD161 (data not shown) receptors remained stable.

### Correlation of NK receptor expressions on decidual NK cells

To assess whether the dNK receptors were linked to each other, we used the Spearman's rank coefficient to measure the statistical correlation between NK receptors whose expression increased or decreased between 8 and 12 weeks of gestation (Table 1). The results show that the percentage of surface expression of NKG2D was inversely correlated to that of CD85j (p = 0.0003) and of KIR2DL1/S1 (p = 0.004). The surface expression of KIR2DL1/S1 correlated positively to that of KIR2DL2/L3/S2 (p = 0.0002) and of CD85j (p<0.0001). Furthermore, the expression of the CD85j inhibitory receptor was correlated to that of KIR2DL2/L3/S2 (p = 0.004). In contrast, the surface expression of NKp30 and NKp44 receptors was not significantly correlated to that of the other receptors (Table 1).

**Table 1:** Spearman correlation coefficient (p-value) of surface expression between NK receptors (n = 28).

	NKp44	CD85j	NKG2D	NKp30	KIR2DL1/S1	KIR2DL2/L3/S2
**NKp44**	1	0. 38 (0.05)	−0.26 (0.18)	0.39 (0.04)	0.28 (0.15)	0.638 (0.04)
**CD85j**		1	**−0.62** * **(0.0003**)	0.13 (0.51)	**0.78 (<0.0001)**	**0.53 (0.004)**
**NKG2D**			1	−0.214 (0.49)	**−0.452 (0.004)**	−0.39 (0.04)
**NKp30**				1	0.27 (0.17)	0.25 (0.20)
**KIR2DL1/S1**					1	**0.65 (0.0002)**
**KIR2DL2/L3/S2**						1

*Bold data are statistically significant (p-value ≤0.004).

### Evolution of decidual NK subpopulations during the first trimester of pregnancy

**Figure 3:**
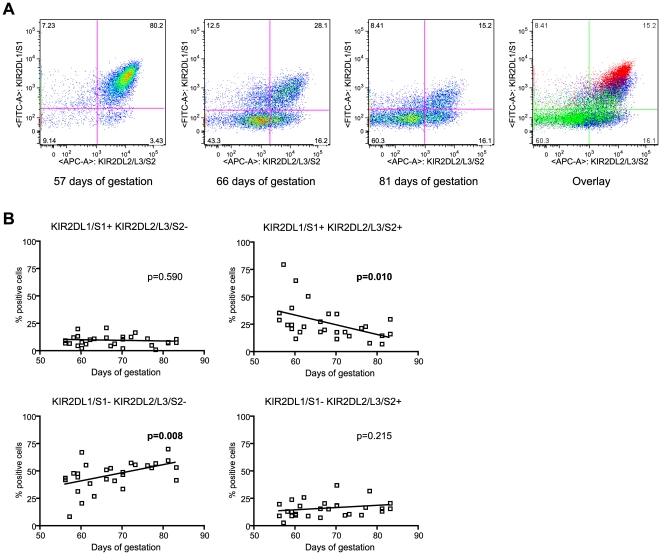
Evolution of the KIR2DL1/S1 - KIR2DL2//L3/S2 dNK subset during the first trimester of pregnancy. Decidual mononuclear cells were analyzed by flow cytometry immediately after isolation. dNK cells were defined as the CD3^−^/CD56^+^ population within the decidual cells. (A) KIR2DL1/S1 and KIR2DL2/L3/S2 co-expression was analyzed on samples taken at 57 days (early, red), 66 days (middle, blue) and 81 days of gestation (late, green). Representative donor analyses are shown individually and overlaid (right dot plot). (B) Linear regression for each subset percentage within the dNK cells (Y) and day of gestation (X). The analyses were performed on decidual cells from 28 different donors, individually represented by a dot.

Because of the strong correlation between KIR2DL1/S1 and KIR2DL2/L3/S2 expression, we analyzed the evolution of the dNK subsets that expressed these markers during the first trimester of pregnancy by flow cytometry (Fig. 3 A & B). The results showed that the majority of KIR2DL1/S1^+^ dNK cells co-expressed the KIR2DL2/L3/S2 receptor at 8 weeks of gestation. During the ensuing weeks, this KIR2DL1/S1^+^/KIR2DL2/L3/S2^+^ dNK subset decreased significantly (p = 0.010), while the proportion of cells that did not express these receptors (KIR2DL1/S1^−^/KIR2DL2/L3/S2^−^ cells) increased significantly (p = 0.008). The percentage of cells of an intermediate phenotype, expressing one or the other receptor (KIR2DL1/S1^+^/KIR2DL2/L3/S2^−^ or KIR2DL1/S1^−^/KIR2DL2/L3/S2^+^) remained stable between 8 and 12 weeks of gestation (p = 0.590 and p = 0.215 respectively).

**Figure 4:**
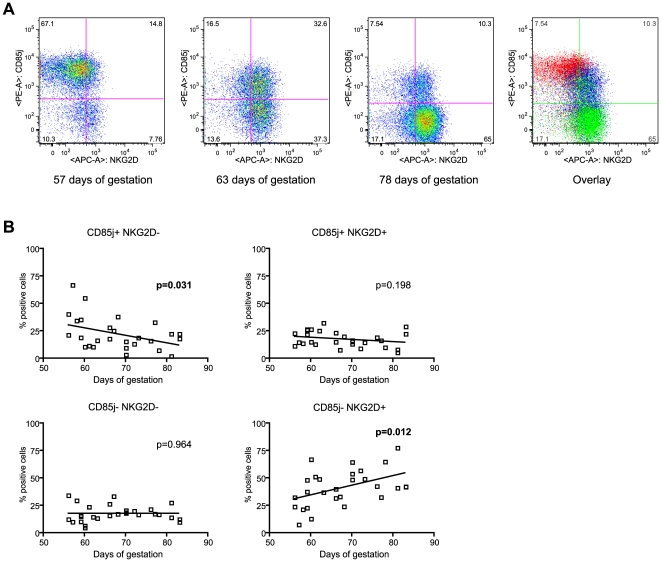
Evolution of the CD85j - NKG2D dNK subset during the first trimester of pregnancy. Decidual mononuclear cells were analyzed by flow cytometry immediately after isolation. The dNK cells were defined by the CD3^−^/CD56^+^ population within the decidual cells. (A) CD85j and NKG2D co-expression was analyzed on samples taken at 57 days (early, red), 63 days (middle, blue) and 78 days of gestation samples (late, green). Representative donor analyses are shown individually and overlaid (right dot plot). (B) Linear regression for each subset, percentage within the dNK cells (Y) and day of gestation (X). The analyses were performed on decidual cells from 28 different donors, individually represented by a dot.

Because of their opposing functions (inhibitory versus activatory, respectively) and their roles in HIV-1 infection [9], [10], we also studied the linked evolution between the CD85j and NKG2D receptors during the first trimester of pregnancy (Fig. 4 A & B). We showed that the majority of dNK cells from early samples (8^th^ week of gestation) expressed the CD85j but not the NKG2D receptor (subset CD85j^+^/NKG2D^−^). Between the 9^th^ and the 10^th^ week of gestation, dNK cells began to express the NKG2D receptor and lose CD85j expression. Finally, the loss of CD85j expression was more pronounced for late samples (11^th^ week of gestation) and the majority of dNK cells became CD85j^−^/NKG2D^+^ at this time (Fig. 4 A). Analysis of the evolution of the four NK subsets (CD85j^+^/NKG2D^−^, CD85j^+^/NKG2D^+^, CD85j^−^/NKG2D^+^, CD85j^−^/NKG2D^−^) confirmed that the subset that expressed only CD85j (CD85j^+^/NKG2D^−^) decreased significantly as pregnancy progressed (p = 0.031)(Fig. 4 B). The subset expressing only the NKG2D receptor (CD85j^−^/NKG2D^+^) increased at the end of the first trimester of pregnancy (p = 0.012). The other subsets (CD85j^+^/NKG2D^+^ and CD85j^−^/NKG2D^−^) remained stable during the first trimester of pregnancy (p = 0.198 and p = 0.964 respectively).

Altogether our results indicate that the phenotype of dNK cells evolves during the first trimester of pregnancy.

### Expression of the NK receptor ligands on the decidual CD14^+^ antigen presenting cells and CD3^+^ T lymphocytes during the first trimester of pregnancy

**Figure 5:**
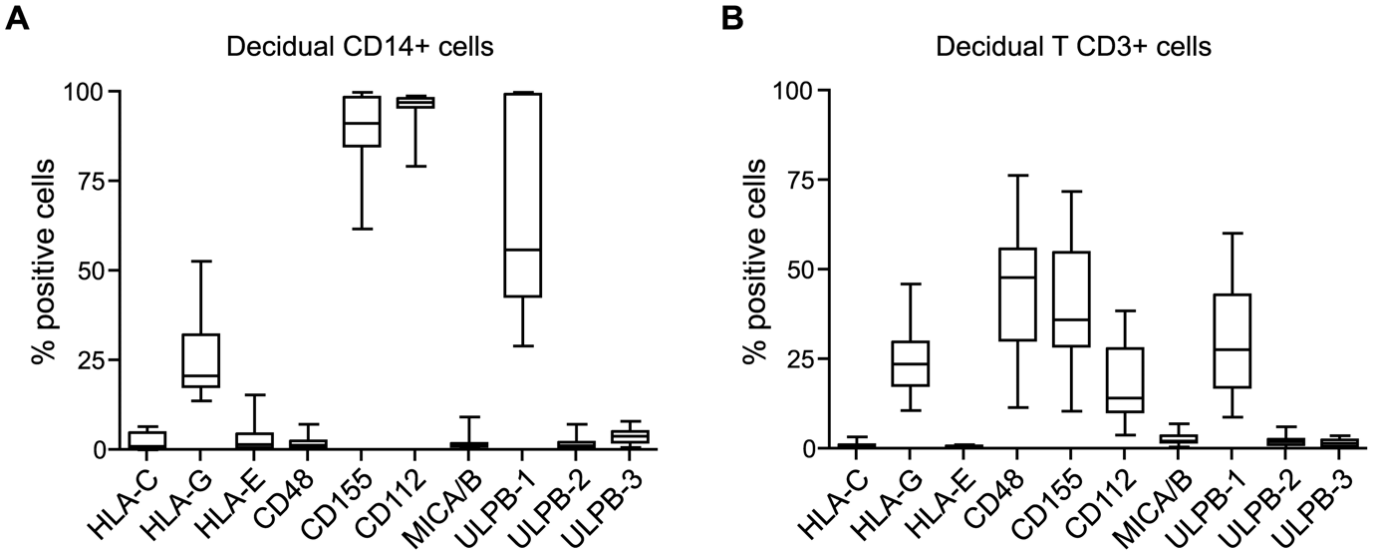
Expression of the dNK receptor ligands at the surface of decidual CD3^+^ and CD14^+^ cells during the first trimester of pregnancy. Decidual mononuclear cells were analyzed by flow cytometry immediately after isolation. The analyses were gated on the CD45^+^/CD3^+^ or on the CD45^+^/CD14^+^ populations. Box-and-whisker plots represent percentages of positive cells for each ligand. Analyses were performed on decidual cells from at least 12 different donors (n = 12–15) between 8 and 12 weeks of gestation.

Because we have previously shown their permissivity to HIV-1 infection [31] and because of their interactions with dNK cells, we analyzed NK receptor ligand expression at the cell surface of the decidual CD14^+^ antigen presenting cells (dAPC) and CD3^+^ T lymphocytes (dLT). To characterize their phenotypes, we performed flow cytometry analysis of 10 ligands on freshly isolated decidual mononuclear cells from the first trimester of pregnancy (n = 13) (Fig. 5, Table 2, Fig. S1). CD48 protein, the ligand of 2B4, was expressed by some dLT cells but was not or weakly detected on dAPC surface as previously described [21]. The dAPC and dLT cells expressed CD155 and CD112, the ligands of DNAM-1, but at different levels (Fig. 5 and Fig. S1). In contrast, the expression of the non-classical MHC class I, HLA-E, the ligand of NKG2A and NKG2C, was not or weakly observed. Some dAPC express at low level HLA-C whereas it was not detected at the dLT surface. We previously described that the pan MHC class I antibody W6/32 recognized all dAPC [31] and dLT cells (data not shown). We observed HLA-G expression, one of the CD85j ligand, on some dAPC and dLT cells. Furthermore, the NKG2D ligands, ULBP-2, ULBP-3 and MICA/B were not expressed at the cell surface of freshly isolated dAPC and dLT cells. However, a large percent of both cell types presented at their cell surface the ligand of NKG2D, ULBP-1.

**Table 2:** Antibodies used for the NK receptor and NK ligand repertoires.

NK receptor Abs	Clone	Ligands	Clone
2B4 (CD244)-PE-Cy5	C1.7 ^(1)^ *	CD48-APC	394607 ^(3)^
CD160-PE	BY55 ^(1)^	HLA-C	L31 ^(4)^
CD85j (ILT-2)-PE	HP-F1 ^(1)^	HLA-G-APC	MEM-G/9 ^(5)^
		HLA-C	L31 ^(4)^
DNAM-1 (CD226)-FITC	DX11 ^(2)^	CD112 (Nectin-2)-PE	R2 477.1 ^(1)^
		CD155 (PVR)-PE	300907 ^(3)^
KIR2DL1/S1 (CD158a/h)-FITC	HP3E4 ^(2)^	HLA-C	L31 ^(4)^
KIR2DL2/L3/S2 (CD158b/j)-APC	GL183 ^(1)^	HLA-C	L31 ^(4)^
KIR2DL4 (CD158d)-FITC	181703 ^(2)^	HLA-G-APC	MEM-G/9 ^(5)^
KIR2DS4 (CD158i)-PE	FES172 ^(1)^	HLA-C	L31 ^(4)^
KIR3DL1/S1 (CD158e)-PE	Z27.3.7 ^(1)^		
NKG2A (CD159a)-APC	131411 ^(3)^	HLA-E	MEM-E/08 ^(5)^
NKG2C (CD159c)-APC	134591 ^(3)^	HLA-E	MEM-E/08 ^(5)^
NKG2D (CD314)-APC	ON72 ^(1)^	MICA/B-APC	159207 ^(3)^
		ULBP1-PE	1708118 ^(3)^
		ULBP2-APC	165903 ^(3)^
		ULBP3	166510 ^(3)^
CD94-FITC	HP3D9 ^(2)^		
CD161-PE-Cy5	DX12 ^(2)^		
CD16-PacificBlue	3G8 ^(1)^		
NKp30 (CD337)-Alexa 647	P30-15 ^(2)^		
NKp44 (CD336)-PE	Z231 ^(1)^		
NKp46 (CD335)-PE	BAB281 ^(1)^		
NKp80-APC	239127 ^(3)^		

*Manufacturer:^ (1)^ BD, ^(2)^ Beckman Coulter, ^(3)^ R&D System, ^(4)^ Kind Gift A.G. Siccardi, ^(5)^ Exbio.

Since expression of dNK receptors evolves during the first trimester of pregnancy, we investigated whether the expression of their respective ligands was modulated during this period. Using linear regression test, we determined that expression of the dNK receptor ligands on dAPC and dLT cell surfaces remained stable on the samples studied (data not shown).

Altogether, these results indicate that ligands of dNK receptors are expressed on dAPC and dLT cell surface at different levels.

## Discussion

In the present study, we provide evidence that the phenotype of dNK cells is dynamic during the first trimester of pregnancy. We first confirmed and strengthened the particularity of the dNK phenotype, by analyzing of the expression of 20 NK receptors at the cell surface. Our results confirmed that dNK cells expressed markers of both CD56^dim^ and CD56^bright^ peripheral blood NK subsets. For the first time, we show that dNK cells express NKp80 and CD161 receptors, and that DNAM-1 receptor is absent from the dNK cell surface. In contrast, most of peripheral blood NK cells express this activatory receptor (data not shown). This marker can thus be used in combination with others to distinguish dNK cells from peripheral blood NK cells.

We observed an evolution of NK receptors at the surface of dNK cells as pregnancy progressed. In fact, the surface expression of KIR2DL1/S1 and KIR2DL2/L3/S2 receptors, NKp30 and NKp44 activatory receptors, and the CD85j (ILT-2) inhibitory receptor significantly decreased with time. In contrast, the surface expression of NKG2D activatory receptor significantly increased during the first trimester of pregnancy. These evolutions were specific to dNK cells, since the expression of these same NK receptors by decidual T CD3^+^ cells (dLT) remained stable (data not shown). The decrease of NK receptor expression could be due to increased interactions with their respective ligands as it has been reported that ligand binding can induce the down-regulation of NK receptors [32], [33]. Indeed, we and others have shown that the ligands of these receptors are effectively expressed at the materno-fetal interface [18], [21], [23]. HLA-C molecule, ligand of KIR2DL1/S1 and KIR2DL2/L3/S2, has been highly detected on placental extravillous trophoblasts [28] and weakly observed in decidual stromal cells [34]. In the present study, we showed that HLA-C is expressed at a low level on CD14^+^ dAPC. Placental trophoblast cells do not express HLA-A and HLA-B [35], however, they express a combination of HLA-C, HLA-E and HLA-G [19], [28], [29]. We detected low expression of HLA-E, the ligand of NKG2A and NKG2C, on dAPC surface. The CD85j receptor binds MHC class I molecules and preferentially interacts with the HLA-G molecule [18]. HLA-G is mainly expressed by placental trophoblast cells and play a crucial role in immunoregulatory functions during pregnancy [36]. The present study showed that HLA-G is detected on some decidual immune cell surface (dAPC and dLT). The ligands of NKG2D were absent from the surface of healthy trophoblast [21]. We observed that ULBP-1 is the only NKG2D ligand expressed at the surface of dAPC and dLT cells. The ULBP-1 expression on decidual immune cells might be related to the cell maturation status [37], [38] and cytokines microenvironment [39] as it has been previously described on peripheral antigen presenting cells. Other studies have shown that the ligands of NKp44 are expressed by trophoblasts [23]. The ligands of NKp30 and NKp44 are also detected within decidual tissue [18]. The binding of anti-NKp30 does not induce a cytolytic activity of dNK cells [40]. Conversely, the binding of anti-NKp46 receptor antibody stimulates the cytolytic activity of dNK cells *in vitro* [40], but the cognate ligands have not been detected at the materno-fetal interface [18]. Moreover, our results show that expression of NKp46 at the dNK cell surface is not modified during the first trimester of pregnancy. dAPC and dLT express CD155 and CD112 whereas their cognate receptor DNAM-1 is not detected at dNK cell surface. Finally, we confirmed that the ligand of 2B4, CD48 protein, is expressed by some decidual CD3^+^ T lymphocytes [21].

A previous study reported a decrease of KIR2DL1/S1 and KIR2DL2/L3/S2 expression at the dNK cell surface between 6 and 12 weeks of gestation [30]. This modulation was observed only for dNK cells and HLA-C binding KIR, and not for peripheral blood NK cells (pNK) and HLA-B binding KIR. However, the authors did not discuss this decrease of expression in their study. It should be noticed that the commercially available antibodies to detect KIR2DL1/S1 and KIR2DL2/L3/S2 do not allow the distinction between the activatory or inhibitory function of these receptors.

At the beginning of the first trimester of pregnancy, the dNK cell activity is mainly regulated by the decidual cells (stromal cells, decidual antigen presenting cells…) through soluble factors and ligand-receptor interactions [21], [34]. We showed, in the present study, that ligands of NK receptors were also expressed by decidual immune cells. Subsequently, the frequency of the ligand-receptor interactions would increase due to extravillous trophoblast invasion. Because of the expression of HLA-C, HLA-G, NKp44-L by trophoblasts, the probability of stimulating the corresponding receptors increases upon trophoblast invasion. The phenotype evolution of the dNK subsets could result from different processes: the downregulation of dNK receptors after ligand binding [32], [33], the phenotypic shift of the dNK population or the clonal expansion of specific dNK subsets. The expression of receptors such as NKp44, CD85j, KIR2DL1/S1 and KIR2DL2/L3/S2 at the dNK cell surface would decrease to ensure the cell activation balance. The activation homeostasis of dNK cells would be preserved, despite variations of the stimulation signals within the decidua during pregnancy. In line to the previously reported modulation of dNK secretion profiles [17], [26], first trimester evolution of NK receptor expression could impact on cytokine and angiogenic factor release. Indeed, previous studies reported that the binding of anti-NKp30, anti-NKp44 or anti-NKG2D antibodies at the dNK cell surface induces the secretion of soluble factors such as IFN-γ, TNF-α, CCL-3, CCL-4 and angiogenic factors [18]. Modulation of dNK receptor expressions would so modify the secretion profile of dNK cells during the pregnancy as noticed by Bulmer's studies [17], [26]. Indeed, dNK cells produce different amounts of angiogenic factors and cytokines during the first trimester of pregnancy, compared to the second [17], [26]. Overall, these studies showed an evolution of dNK cell functions during pregnancy. Moreover, the increase of NKG2D expression may help to protect the fetus against pathogenic infections, since the NKG2D ligands, MICA/B and ULBP1-5, are mainly induced by cellular stresses such as cell damage or pathogen infection [41], [42]. The dNK cells could have an antiviral activity against infected decidual cells during the pregnancy mediated through cytolysis or through soluble factors such as cytokines and chemokines. Earlier in the first trimester of pregnancy, when NKG2D is not expressed on dNK cells, antiviral activity could be mediated by CD85j as it has been previously described on peripheral NK cells [9], [10].

In conclusion, in this study, we have shown that the phenotype of dNK cells is dynamic during the first trimester of pregnancy. Evolution of NK receptor expression could preserve the activation homeostasis of dNK cells. Our results highlight for the first time an evolution of the dNK cell phenotype from CD85j^+^ to NKG2D^+^. These two receptors are known to mediate antiviral activity of NK cells in the periphery by distinct mechanisms [9], [10]. Even if there is no functional evidence in the literature for a sentinel role of dNK cells against pathogens, a role of dNK cells in the control of pathogen transmission has repeatedly been suggested [43], [44]. However, in addition to their known builder function [12], dNK cell antiviral activities still need to be demonstrated.

## Methods

### Ethics statement

All the women donors in this study provided their written informed consent. The study was approved by the ethics committee of the Hôtel Dieu (Paris, France), the Assistance Publique des Hôpitaux de Paris (n° VAL/2006/06-41/01), the Agence de Biomédecine (n° PFS08-013) and the biomedical research committee of the Institut Pasteur, Paris, France (n° RBM/2005.024).

### Human decidual tissue collection

Decidual tissue samples were obtained from 43 healthy women who underwent voluntary pregnancy termination during the first trimester of pregnancy (8–12 weeks of gestation) at the Antoine Béclère Hospital, Clamart, France or the Tenon Hospital, Paris, France. Samples used for the NK repertoire were distributed as followed: 8–9 weeks of gestation (n = 11), 9–10 weeks of gestation (n = 5), 10–11 weeks of gestation (n = 6) and 11–12 weeks of gestation (n = 6). Samples used for the NK ligand repertoire were distributed as followed: 8–9 weeks of gestation (n = 3), 9–10 weeks of gestation (n = 3), 10–11 weeks of gestation (n = 6) and 11–12 weeks of gestation (n = 3). Decidua basalis were separated from the other tissues, collected, and then washed using RPMI 1640 Glutamax medium (Gibco, Invitrogen, Cergy Pontoise, France) supplemented with 10% fetal calf serum (PAA, Les Mureaux, France) to eliminate blood and placental contaminants.

### Isolation of decidual mononuclear cells

The freshly isolated decidual basalis tissue was minced and digested under agitation for 1 hour at 37°C, in RPMI 1640 (Gibco) with 1 mg/ml collagenase IV (Sigma, St Quentin Fallavier, France) and 50 U/ml recombinant DNase I (Roche, Meylan, France). The cell suspension was filtered successively through 100, 70 and 40 µm sterile nylon cell strainers (BD Biosciences). The mononuclear cell population was isolated using Lymphocyte Separation Medium (PAA).

### Antibodies

The following antibodies were used for the gating of decidual immune cells: Amcyan labelled anti-CD45 (2D1); PacificBlue-labeled anti-CD14 (M5E2); FITC-conjugated anti-CD69 (FN50); PE-TexasRed-labeled anti-CD3 (UCHT1); Alexa700-labeled anti-CD56 (B159). A donkey anti-mouse IgG-PE was used for the indirect staining (Jackson ImmunoResearch Europe Ltd) and isotype matched Ig were used as controls. The antibodies used for the NK receptor and the NK ligand repertoires were presented Table 2.

### NK receptor and NK ligand repertoire analysis on decidual cells

Decidual mononuclear cells were directly stained for surface markers after isolation and subsequently fixed with 1% paraformaldehyde solution (EMS, Hatfield, USA). FACS data were acquired using a LSRII 2-Blue 2-Violet 3-Red 5-Yelgr laser configuration (BD Biosciences). Flow cytometry analyses were performed by Diva (BD Biosciences) and FlowJo 9.0.1 (Tristar) software. For the NK receptor repertoire, analyzed cells were gated on lymphocyte populations that had been defined by FSC/SSC, then NK cells were defined as CD56^+^/CD3^−^ cells. At least 10 000 events in the CD56^+^/CD3^−^ (NK cell) gate were analyzed in each test. For the NK ligand repertoire, dAPC were defined as CD45^+^/CD14^+^ cells and dLT cells as CD45^+^/CD3^+^ cells. At least 1 000 events in the dAPC and dLT cell gates were analyzed.

### Statistical analysis

Linear regression tests were performed to assess the correlation of NK receptor and NK ligand variations over the progression of pregnancy. A p value of <0.05 was considered to be statistically significant.

Two by two correlations between NK receptors for which the expression significantly increased or decreased between 8 and 12 weeks of gestation were estimated with the Spearman coefficient. Adjustments for multiple tests were performed using the method proposed by Benjamini and Hochberg (1995), which controls the False Discovery Rate (FDR). Correlation coefficients were considered to be statistically significant when the associated p value was ≤0.004. Statistical analyses were performed using Prism 4 software (Graph Pad).

## Supporting Information

Figure S1 **Cell surface expression of dNK receptor ligands on decidual immune cells.** Decidual CD14^+^ cells (A) and decidual CD3^+^ cells (B) were stained with Allophycocyanin-conjugated (CD48, ULBP-2, HLA-G, MICA/B), Phycoerythrin-conjugated (CD155, CD112, ULBP-1), or unconjugated (HLA-C, HLA-E, ULBP-3) mAb (grey histograms). Unstained cells or PE-conjugated goat anti-mouse IgG stained cells were used as controls (dotted lines). The numbers indicate the x-fold increase in mean fluorescence intensity over the control. This experiment is representative of n = 12 decidual samples.
(TIF)
