# Supplementary figures and images for: Dynamic Shift from CD85j/ILT-2 to NKG2D NK Receptor Expression Pattern on Human Decidual NK during the First Trimester of Pregnancy
Source: PLoS One. 2012 Jan 5;7(1):e30017. doi: 10.1371/journal.pone.0030017 (PMC3252358; doi:10.1371/journal.pone.0030017)

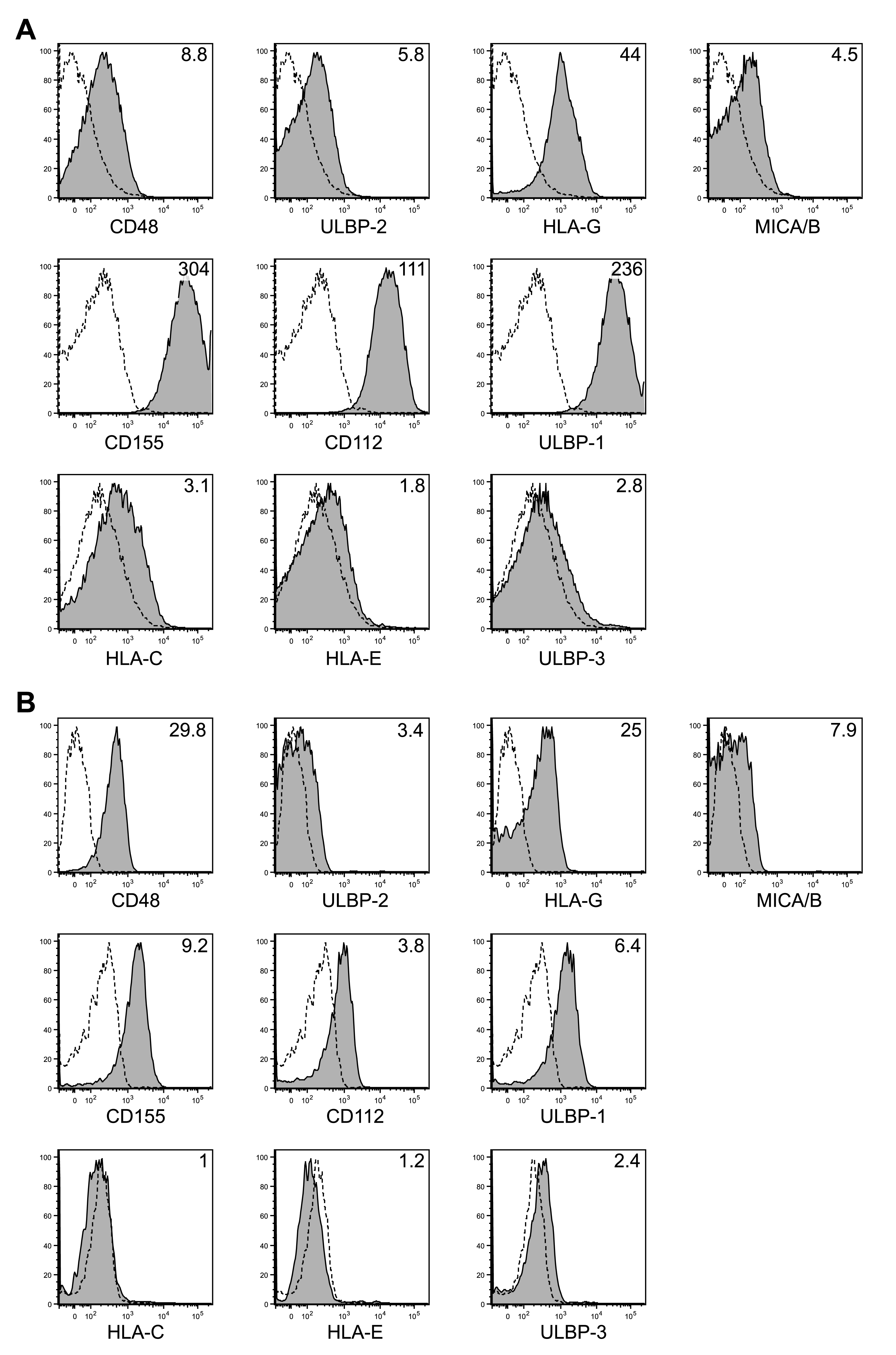

Supplement: Figure S1 — Cell surface expression of dNK receptor ligands on decidual immune cells. Decidual CD14+ cells (A) and decidual CD3+ cells (B) were stained with Allophycocyanin-conjugated (CD48, ULBP-2, HLA-G, MICA/B), Phycoerythrin-conjugated (CD155, CD112, ULBP-1), or unconjugated (HLA-C, HLA-E, ULBP-3) mAb (grey histograms). Unstained cells or PE-conjugated goat anti-mouse IgG stained cells were used as controls (dotted lines). The numbers indicate the x-fold increase in mean fluorescence intensity over the control. This experiment is representative of n = 12 decidual samples. (TIF) [file pone.0030017.s001.tif]
